# Maternal urinary phthalates and sex-specific placental mRNA levels in an urban birth cohort

**DOI:** 10.1186/s12940-017-0241-5

**Published:** 2017-04-05

**Authors:** Jennifer J. Adibi, Jessie P. Buckley, Myoung Keun Lee, Paige L. Williams, Allan C. Just, Yaqi Zhao, Hari K. Bhat, Robin M. Whyatt

**Affiliations:** 1 Department of Epidemiology, Graduate School of Public Health, University of Pittsburgh, 130 Desoto Street, Parran Hall 5132, Pittsburgh, PA 15261 USA; 2 Department of Obstetrics, Gynecology and Reproductive Sciences, University of Pittsburgh, Pittsburgh, USA; 3 Department of Environmental Health and Engineering, Johns Hopkins Bloomberg School of Public Health, 615 N. Wolfe Street, Baltimore, MD 21205 USA; 4 Department of Biostatistics, Harvard T.H. Chan School of Public Health, 665 Huntington Avenue, Building I, Room 415, Boston, MA 02115 USA; 5 Department of Preventive Medicine, Icahn School of Medicine at Mount Sinai, One Gustave L. Levy Place, Box 1057, New York, NY 10029 USA; 6 Division of Pharmacology and Toxicology, UMKC School of Pharmacy, University of Missouri-Kansas City, 2464 Charlotte Street, HSB 5251, Kansas City, MO 64108 USA; 7 Department of Environmental Health Sciences, Mailman School of Public Health, 722 W 168th Street, New York, NY 10032 USA

**Keywords:** Placenta, Phthalates, mRNA, Sex difference, *CGA*, Correlated vector

## Abstract

**Background:**

Prenatal urinary concentrations of phthalates in women participants in an urban birth cohort were associated with outcomes in their children related to neurodevelopment, autoimmune disease risk, and fat mass at 3,5,7, and 8 years of life. Placental biomarkers and outcomes at birth may offer biologic insight into these associations. This is the first study to address these associations with candidate genes from the phthalate and placenta literature, accounting for sex differences, and using absolute quantitation methods for mRNA levels.

**Methods:**

We measured candidate mRNAs in 180 placentas sampled at birth (*HSD17B1, AHR, CGA, CYP19A1, SLC27A4, PTGS2, PPARG, CYP11A1*) by quantitative PCR and an absolute standard curve. We estimated associations of log_e_ mRNA with quartiles of urinary phthalate monoesters using linear mixed models. Phthalate metabolites (*N* = 358) and mRNAs (*N* = 180) were transformed to a z-score and modeled as independent, correlated vectors in relation to large for gestational age (LGA) and gestational diabetes mellitus (GDM).

**Results:**

*CGA* was associated with 4 out of 6 urinary phthalates. *CGA* was 2.0 log_e_ units lower at the 3^rd^ vs. 1^st^ quartile of mono-n-butyl phthalate (MnBP) (95% confidence interval (CI): −3.5, −0.5) in male placentas, but 0.6 log_e_ units higher (95% CI: −0.8, 1.9) in female placentas (sex interaction *p* = 0.01). There was an inverse association of MnBP with *PPARG* in male placentas (−1.1 log_e_ units at highest vs. lowest quartile, 95% CI: −2.0, −0.1). *CY19A1*, *CYP11A1, CGA* were associated with one or more of the following in a sex-specific manner: monobenzyl phthalate (MBzP), MnBP, mono-iso-butyl phthalate (MiBP). These 3 mRNAs were lower by 1.4-fold (95% CI: −2.4, -1.0) in male GDM placentas vs. female and non-GDM placentas (*p*-value for interaction = 0.04). The metabolites MnBP/MiBP were 16% higher (95% CI: 0, 22) in GDM pregnancies.

**Conclusions:**

Prenatal concentrations of certain phthalates and outcomes at birth were modestly associated with molecular changes in fetal placental tissue during pregnancy. Associations were stronger in male vs. female placentas, and associations with MnBP and MiBP were stronger than other metabolites. Placental mRNAs are being pursued further as potential mediators of exposure-induced risks to the health of the child.

**Electronic supplementary material:**

The online version of this article (doi:10.1186/s12940-017-0241-5) contains supplementary material, which is available to authorized users.

## Background

Urinary phthalate metabolites, which are detectable in 99–100% of residents in the United States, enter our bodies through dietary ingestion [1, 2] inhalation [3], and dermal uptake [4]. Exposure levels vary by age, race, and income [5–7]. Urinary phthalate concentrations in pregnant women have been associated with short-term effects on their pregnancies (timing of labor, biomarkers related to risk of preeclampsia) [8–10] and with long-term effects in their children (future reproductive function, asthma and eczema, fat mass, neurocognitive and neurobehavioral outcomes) [11–13]. The role of the human placenta and the mechanisms that might explain these effects are not well studied or understood.

Consideration of the placenta can grant temporal and spatial precision to estimates of association given the placenta’s physical location between maternal circulating phthalates and the developing embryo/fetus at precisely the time when organs are developing and cells and tissues are undergoing programming. With this knowledge, we can develop better measures of the placental response to the exposure, and potentially within an early enough timeframe to alter the short-term risks to the pregnancy and the long-term risks to the child.

Our pilot study demonstrated associations between maternal urinary phthalates and placental mRNAs, but did not account for sex differences in these associations [14]. Two in vitro studies measured mRNA changes in human placental cells in response to doses of mono-2-ethylhexyl phthalate (MEHP) [15, 16]. These studies did not account for sex differences and the dose ranges were 50–200 fold higher than what are measured in the urine of pregnant women. Our aim was to analyze these relationships in the context of commonly measured phthalate levels in pregnant women, and to account for sex differences.

The recent emphasis on sex differences in placental responses (reviewed in [17]) underlines the fact that the placenta is fetal, and not maternal, tissue in its origin, function, and molecular profile. There is a need currently for human placental data and unifying theories as to how the molecular machinery of a female vs. a male placenta might differ at baseline, behave differently in response to a chemical exposure, and differentially alter fetal development. Two cellular mechanisms, one involving autophagy and another involving receptor isoforms, have been identified that explain why the response to a maternal exposure (adiposity, cortisol) might differ between a female vs. a male placenta, i.e. effect modification by sex [18, 19].

There are similarly reasons to suspect that the placenta may play a different role in regulating aspects of fetal development in a male vs. a female [20]. Canonical placental functions (nutrition, gas exchange, etc.) play an important role after 10 weeks of pregnancy, or the end of organogenesis [21]. However, prior to this point, the placenta may be synthesizing and secreting molecules necessary for proper, sex-specific, tissue differentiation [22].

We measured the association of 6 maternal urinary phthalate metabolite concentrations in late pregnancy with the mRNA concentrations of 8 candidate genes in placental tissue biopsied at birth. These placentas were sampled from the same pregnancies in which associations have been reported between prenatal phthalate concentrations and indicators of neurodevelopment at 3 and 7 years of age [23, 24]; with the risk of asthma at 8 years of age [25]; and the risk of obesity from 4 to 7 years [26, 27]. These candidate genes were chosen based on *a priori* knowledge of the transcriptional effects of phthalates, their expression in the human placenta (reviewed in [14]), and associations with placentally-mediated pathologies such as preeclampsia (*HSD17B1, CYP19A1, CGA, AHR*) [28–31], gestational diabetes (*PPARG, CGA*) [29, 32], fetal growth restriction (*AHR*) [28], and fetal programming (*SLC27A4*) [33]. A secondary aim was to evaluate the independent relationships of the phthalates and placental biomarkers with these types of outcomes at birth (fetal size, gestational diabetes).

## Methods

### Subjects

African American and Dominican American pregnant women (*N* = 358) were enrolled in the phthalate substudy of the Mothers and Newborns Study of the Columbia Center for Children’s Environmental Health (CCCEH) [34]. A subset of these women donated placental tissue at birth (180). They delivered at one of three community hospitals in New York City between 2002 and 2006. The present study is a full analysis of the pilot placenta study previously described [14]. CCCEH participants gave informed consent to participation including collection of urine, sampling of the placenta at delivery, and access to prenatal and birth records. Maternal and pregnancy characteristics and birth/neonatal outcomes were derived from the study prenatal questionnaire, the mother’s medical record, and/or the baby’s birth record. Large for gestational age (LGA) was calculated with U.S. reference birth weight values by race/ethnicity and sex. [35] Gestational diabetes was ascertained by combined self-report from the prenatal questionnaire in the third trimester and from the birth record given potential inconsistencies across reporting on the birth record at the three hospitals. The CCCEH study and subsequent data analysis was approved by the Columbia University Medical Center Institutional Review Board (IRB) and the University of Pittsburgh IRB.

### Placental samples

Biopsies were taken from the fetal side of the placenta by cutting into the chorionic plate and sampling of fetal chorionic villi, and preserved in RNALater (Qiagen, USA). To capture variability in gene expression within the placenta, one sampling site was close to the umbilical cord insertion point and one close to the outer margin of the placenta [36]. We generated gene expression data for 2 biopsies from each of 164 placentas. For some placentas, either tissue was unavailable or the tissue quality and/or quantity were insufficient for analysis, thus, in 12 placentas we relied on 1 biopsy, while we had more than 2 biopsies from 4 placentas. Samples were preserved in RNALater (Qiagen, USA) and stored at -80C. Based on histologic analysis, there was minimal contamination of the biopsies with cells from the chorionic membrane and basal plate [36].

### RNA from placental tissue biopsies

Total RNA was isolated from each tissue sample in two batches. The first batch of 55 placentas was analyzed as described previously [14]. The second batch was analyzed using automated methods. Tissue was homogenized using the Tissue Lyser (Qiagen, USA) according to the supplier’s protocol. RNA isolation was carried out using the QIAcube (Qiagen, USA). RNA quality was assessed by separation and visualization of the 28s and 18s ribosomal RNA bands on an analytical agarose gel and quantitated by spectrometry. RNA was reverse transcribed to cDNA using Superscript II (Life Technologies, USA).

### Gene expression in placental tissue biopsies

We selected 8 candidate genes whose expression has been shown in prior experimental studies to be affected by one or more of the 6 phthalates analyzed, and chorionic gonadotropin alpha [*CGA*] was added because of its specificity to the human placenta and known variability across placental tissue [37]. The candidate genes (17beta hydroxysteroid dehydrogenase type 1 [*17BHSD1*]*,* arylhydrocarbon receptor [*AHR*]*,* aromatase [*CYP19A1*]*, CGA,* P450 cholesterol side chain cleavage enzyme [*CYP11A1*]*,* peroxisome proliferator activated receptor gamma [*PPARG*]) and details of the qPCR primers were described in detail previously [14]. A transcript of 114 base pairs (bp) for the fatty acid transport protein 4 (*SLC27A4*) was added [38], as well as a 204 bp transcript for COX2 [*PTGS2*]. *PTGS2* was only measured in 124 of the samples. 18 s (*RN18S1*) mRNA was measured in all samples as an internal control. Plates were run sequentially on the ABI 7900HT quantitative polymerase chain reaction analyzer (Applied Biosystems, USA). Each sample was run in duplicate, and values not falling within 50% of their mean were rerun. Specificity of the PCR product was evaluated using the melting curve and by running a 2% agarose gel to visualize the PCR product. Absolute quantitation of mRNA concentration in the original sample was achieved using a standard curve generated for each batch, as described previously [39]. The R^2^ for the standard curve was between 0.98 and 1.00; the plate was rerun if it was < 0.95. mRNA concentrations were log-transformed before analysis.

### Urinary phthalates

Maternal urine samples were collected from participants at a mean gestational age of 34 weeks (standard deviation 3.0 weeks). Phthalates were measured at the Centers for Disease Control and Prevention and included a panel of phthalate metabolites, of which we used the 6 most frequently detected monoesters, i.e., mono-n-butyl phthalate (MnBP, 100%), monobenzyl phthalate (MBzP, 100%), mono-2-ethylhexyl phthalate (MEHP, 84%), monoethyl phthalate (MEP, 100%), and mono-iso-butyl phthalate (MiBP, 99%). In a separate analysis, we used the following oxidative metabolites of di-2-ethylhexyl phthalate: mono-2-ethyl-5-oxohexyl (MEOHP, 100%), mono-2-ethyl-5-hydroxyhexyl phthalate (MEHHP, 100%), mono-2-ethyl-5-carboxypentyl phthalate (MECPP, 100%); and an oxidative metabolite of di-n-butyl, di-iso-butyl and other higher molecular weight phthalates, mono-3-carboxy-propyl phthalate (MCPP, 94%) [40, 41]. The analysis involved enzymatic deconjugation of phthalate glucuronides, solid-phase extraction, separation by HPLC, and detection by isotope-dilution tandem mass spectrometry [42]. The limits of detection (LODs) were MEHP, 3.2 nM; MnBP, 1.8 nM; MBzP, 0.43 nM; MEP, 2.1 nM; MiBP, 1.0 nM; MEOHP, 1.5 nM; MEHHP, 1.1 nM; MECPP, 0.8 nM; MCPP, 0.6 nM. To analyze the DEHP oxidative metabolites, we summed the molar quantities of MEOHP, MEHHP, and MECPP (DEHP-oxo). For statistical analysis, concentrations below the LOD were assigned a value equal to the LOD divided by the square root of two. Specific gravity was measured using a PAL 10-S hand-held refractometer (Atago, USA).

### Statistical analysis

#### Association with placental tissue mRNAs

Urinary phthalate nM concentrations were modeled as quartiles and gene expression values were natural log-transformed before analysis. To estimate associations between maternal urinary phthalate concentrations and log_*e*_ mRNA levels, we fit mixed effects models to account for the correlation of multiple samples within a placenta. We simultaneously included multiple phthalates in all models for two reasons: a) to control for inter-individual differences in phthalate metabolism and excretion, and b) to estimate independent effects of the 5 phthalate monoesters. MnBP/MiBP, MBzP, MEHP, and MEP estimates were adjusted for each other. MnBP and MiBP were each modeled separately given their high correlation (*r* = 0.77). Phthalate metabolite formation proceeds as: phthalate diester > hydrolysis by non-specific lipases > *phthalate monoester metabolites*> > activation of polymorphic enzymes in the liver and placenta to metabolize and excrete xenobiotics> > *phthalate oxidative metabolites* [43]. In the case of pregnancy, this transformation process may be confounded by maternal genetics, health and placental function, rendering the oxidative metabolites a suboptimal proxy for DEHP:MEHP exposure and biologic action [44]. Combining the two types of metabolites into a single molar sum may reduce precision in the DEHP:MEHP exposure estimate. To estimate associations with monoesters, we did not adjust for the downstream oxidative metabolites. To estimate correlations of the oxidative metabolites and gene expression, we used the molar sum of the DEHP oxidative metabolites (DEHP-oxo) and co-adjusted for the monoesters. To rule out potential multiple co-linearity of the single measures of phthalate metabolites in relation to a mean gene expression per placenta, we fit linear regression models and examined the variance inflation factor and the condition index output as co-linearity diagnostics. Models were adjusted for 18s mRNA concentration to control for variability in RNA quality and quantity going into the reaction, as well as a term for technician who carried out the sampling, the qPCR batch, urinary dilution (z-score of specific gravity), sex of the neonate and body mass index (BMI) at the start of pregnancy. The types of confounding that were evaluated include maternal hydration and kidney function (i.e. specific gravity) [45], maternal adiposity and weight, time elapsed between delivery and placental tissue sampling, maternal demographics (race, education, marital status, income), maternal medical and reproductive history, year of delivery, and maternal medications and supplements. Sex of the neonate was imputed for the 2 missing subjects, based on placental hormone gene expression values. For the analysis of associations between urinary phthalates and outcomes in the full sample, we evaluated the following types of confounding: year and season of birth, maternal size and weight gain, maternal demographics, smoking, chronic disease, and reproductive history. Variables that were significantly correlated with phthalates in the presence of the birth outcome variable were retained in the model. If there was 10% or greater missingness in a variable, it was excluded from the analysis. Given the larger sample size, we allowed for small differences in covariates by model in order to address differences in sources of exposure and biologic action between the metabolites. For each phthalate and gene association, we fit a model to calculate the *P*-value for the interaction of phthalate concentrations and sex of the placenta. Using parameters from this model, we computed sex-specific beta coefficients and confidence intervals with quartile 1 as the referent within sex, and female quartile 1 as the referent for male quartile 1.

#### Associations with birth outcomes

We estimated associations of urinary phthalate biomarkers with LGA and GDM outcomes in the full cohort (*N* = 358), and mRNA biomarkers with the same outcomes in the placenta substudy (*N* = 180). We compared biomarker mean values across the outcomes, instead of calculating odds ratios given the relatively small sample sizes for rare outcomes. For the 2 urinary phthalate metabolites that originate from DnBP and DiBP (MnBP, MiBP), and separately for DEHP-oxo and MCPP, z-score means were modeled as a correlated vector of responses within each subject, adjusting for phthalate concentrations (MBzP, MEHP, MEP) and other covariates (18s mRNA, qPCR batch, technician, sex of the baby and BMI). The mixed effects model approach allows for the analysis of the combined mean phthalate concentration while also taking into account differences between phthalates, and simultaneously adjusting for the within person phthalate correlations [46]. We assumed equal correlation between any two phthalates measured within a single person. This approach has been used in the setting of factor analysis, where multiple exposures or biomarkers are first standardized as z-scores to put them on the same scale, and then factors are derived based on the inter-correlation among these standardized measures of exposures [47]. To estimate differences in the other 3 phthalates (MBzP, MEHP, MEP) that originate from different phthalate diesters, we modeled them as individual metabolites.

In the subset with placental mRNA data (*N* = 180), we applied a similar strategy to estimate correlations between placental mRNAs and birth outcomes that relate to placental function, independently of phthalates, and additionally adjusted for placental-fetal sex, urinary dilution, 18s RNA mRNA (housekeeping gene), body mass index at the beginning of pregnancy, qPCR batch, technician, urinary dilution, and year of sample. We estimated percent change between the cases and the non-cases using the model intercept and the beta coefficient for the outcome. We did not adjust for multiple comparisons given the exploratory nature of the analysis. Analyses were carried out using SAS 9.2 (SAS, USA).

## Results

The 180 placenta donors from CCCEH generally resembled the larger cohort of 358 women with urinary phthalate measures, from which they were sampled [3] (Table 1); yet they also differed from the larger sample as 1) the mothers carrying females had lower BMI’s than the mothers carrying males, 2) there was a lower proportion of African-American vs. Dominican participants, and 3) mothers carrying males had a higher prevalence of LGA births than mothers carrying females. The geometric mean phthalate monoester concentrations were (highest to lowest): MEP (1.3 μM) > MnBP (174 nM) > MBzP (81 nM) > MiBP (48 nM) > MEHP (18 nM).

**Table 1:** Characteristics of the Columbia Center for Children’s Environmental Health (CCCEH) placenta and phthalate substudies

	Placenta substudy (*N* = 180)		Phthalate study (*N* = 358)	
	Mean (SD)		Mean (SD)	
	Female	Male	Female	Male
Maternal age (yr)	26.0 (4.9)	25.9 (4.5)	25.4 (4.9)	25.6 (4.8)
Gestational age at delivery (wks)	39.4 (1.6)	39.4 (1.6)	39.5 (1.5)	39.4 (1.5)
Body mass index at the start of pregnancy	25.8 (6.8)	27.3 (7.3)	26.0 (6.6)	26.8 (7.6)
	N (%)		N (%)	
	Female	Male	Female	Male
N	90 (50)	90 (50)	189 (53)	169 (47)
Vaginal births	63 (73)	56 (67)	137 (75)	112 (70)
Parity				
0 livebirths	35 (39)	39 (44)	87 (44)	72 (43)
1 livebirths	39 (44)	28 (31)	62 (32)	54 (32)
> 1 livebirths	15 (17)	22 (25)	43 (23)	43 (25)
Ethnicity				
Dominican	67 (74)	73 (82)	130 (69)	121 (72)
African American	23 (26)	16 (18)	59 (31)	48 (28)
Education				
< High school	4 (4)	7 (8)	6 (3)	10 (6)
High school or equivalent exam	62 (69)	56 (63)	140 (74)	112 (66)
> High school	24 (27)	26 (29)	43 (23)	47 (28)
Marital status				
never married	65 (72)	50 (56)	134 (72)	102 (60)
widowed, divorced, separated	5 (6)	9 (10)	8 (4)	13 (8)
married	20 (22)	30 (34)	47 (25)	54 (32)
Large for gestational age	3 (4)	11 (13)	13 (7)	15 (9)
Gestational diabetes	6 (7)	8 (9)	11 (6)	12 (7)

Of the 40 tests to identify main effect associations of urinary phthalate monoester quartiles and placental mRNA, 2 tests (5%) reached statistical significance at *p* < =0.05: MiBP and *PTGS2*, MEP and *PPARG* (data not shown). In the 40 tests to assess phthalate and sex interactions, 8 (20%) reached statistical significance at *p* < 0.05.

**Fig. 1:**
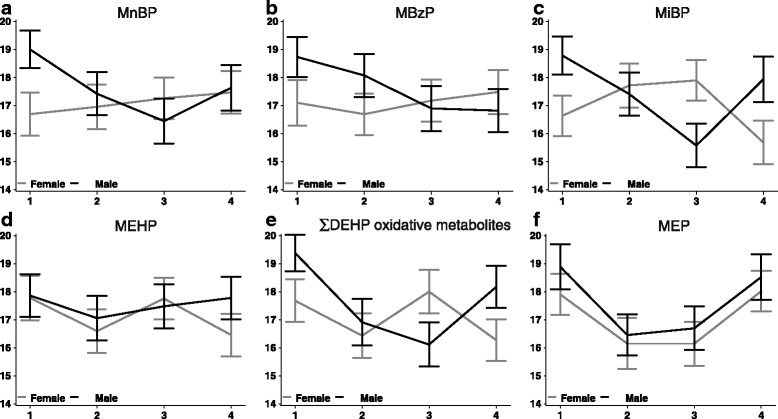
Sex-specific associations of maternal urinary phthalate metabolites, expressed as quartiles, with log_e_ chorionic gonadotropin alpha (CGA) mRNA expression (mean, SE) in placental biopsies sampled at delivery. **a** Mono-n-butyl phthalate **b** Monobenzyl phthalate **c** Mono-iso-butyl phthalate **d** Mono-2-ethylhexyl phthalate **e** Molar sum of DEHP oxidative metabolites **f** Monoethyl phthalate

**Fig. 2:**
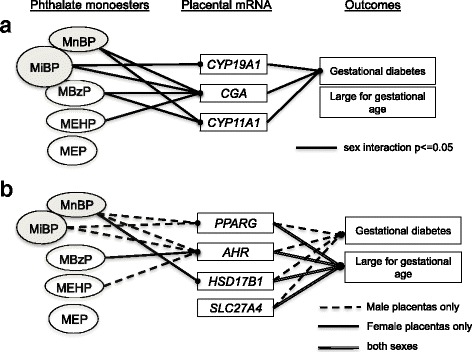
Summary of the reported associations. **a** Associations of phthalate monoesters, placental mRNAs (sex-specific), and outcomes. **b** Associations of phthalate monoesters with placental mRNAs in male pregnancies only or in female pregnancies only, and associations of placental mRNAs with outcomes. Shaded metabolites and the 2 groups of genes were modeled as correlated vectors. MnBP and MiBP concentrations were 16% higher (95% CI 0, 32) in GDM pregnancies vs. non-GDM pregnancies

*CGA* mRNA was associated with 4 of the 5 monoester phthalates, and also with DEHP-oxo (Table 2, Additional file 1: Table S1). Associations differed significantly by sex for three of the genes: *CGA* (Fig. 1), *CYP19*, and *CYP11A1* (Table 2, Fig. 2). MnBP associations with *CGA* mRNA were opposite in direction between males and females. *CGA* expression was 2.0 log_e_ units lower (95% CI: −3.6, −0.5) in male placentas at the 3^rd^ quartile (175–357 nM) of MnBP, compared to the first quartile (0–95 nM). This translates as an 82% decrease (95% CI: −144, −20). In the female placentas, *CGA* expression was 0.6 log_e_ units higher in the 3rd vs. the 1^st^ quartile, but the confidence interval included zero (95% CI: −0.8, 1.9). This translates as a 24% increase (95% CI: −30, 78, p-value for sex interaction = 0.01). Results for the 4 mRNAs that did not differ by sex in their phthalate associations are reported in Additional file 2: Table S2. The associations were overall stronger in male placentas than in females, except for *17BHSD1*. In the male placentas, gene expression was generally lower with higher phthalate concentration, except for *SLC27A4* (fatty acid transport protein 4). Other strong inverse, exposure-response associations were observed for MiBP and *CGA* and MnBP and *PPARG* in male placentas. These were of similar magnitude as reported above: 64-77% decrease from the first quartile of exposure. Alternative approaches (log linear, quintiles, spline regressions) for considering phthalate concentrations did not change the conclusions.

**Table 2:** Associations of placental gene expression in female and male placentas with maternal urinary monoester phthalates

	β (Females) (95% CI)	β (Males) (95% CI)	Sex by phthalate interaction *P*-value	β (Females) (95% CI)	β (Males) (95% CI)	Sex by phthalate interaction *P*-value
	*CYP19A1*			*AHR*		
MnBP^a^			0.08			0.19
Sex Q1	ref	1.43 (0.32, 2.54)		ref	1.15 (0.34, 1.97)	
MnBP Q1	ref	ref		ref	ref	
MnBP Q2	0.40 (−0.74, 1.54)	−0.66 (−1.93, 0.61)		−0.17 (−1.14, 0.80)	−1.03 (−2.04, −0.01)	
MnBP Q3	0.22 (−1.14, 1.57)	−0.97 (−2.36, 0.42)		−0.80 (−2.02, 0.42)	−1.62 (−2.81, −0.43)	
MnBP Q4	0.59 (−0.87, 2.05)	−1.80 (−3.90, 0.30)		−0.22 (−1.46, 1.03)	−1.69 (−3.18, −0.19)	
MBzP^b^			0.07			0.22
Sex Q1	ref	0.42 (−0.97, 1.81)		ref	0.75 (−0.29, 1.79)	
MBzP Q1	ref	ref		ref	ref	
MBzP Q2	−0.57 (−1.66, 0.51)	0.11 (−1.44, 1.66)		0.16 (−1.01, 1.33)	0.25 (−0.92, 1.41)	
MBzP Q3	0.35 (−0.92, 1.61)	0.31 (−1.26, 1.89)		0.93 (−0.11, 1.97)	0.51 (−0.66, 1.68)	
MBzP Q4	0.96 (−0.32, 2.24)	−0.34 (−2.02, 1.34)		1.39 (0.17, 2.61)	0.19 (−1.03, 1.41)	
MEHP^c^			0.46			0.90
Sex Q1	ref	0.53 (−0.86, 1.92)		ref	0.69 (−0.29, 1.66)	
MEHP Q1	ref	ref		ref	ref	
MEHP Q2	0.25 (−1.02, 1.52)	−0.09 (−1.11, 0.94)		0.19 (−0.89, 1.27)	−0.20 (−1.13, 0.73)	
MEHP Q3	0.11 (−1.06, 1.28)	−0.78 (−2.00, 0.44)		−0.53 (−1.50, 0.43)	−1.02 (−2.02, −0.02)	
MEHP Q4	0.09 (−1.35, 1.53)	0.24 (−0.86, 1.34)		−0.12 (−1.24, 1.00	−0.53 (−1.43, 0.38)	
MEP^d^			0.89			0.52
Sex Q1	ref	0.38 (−0.68, 1.44)		ref	0.39 (−0.59, 1.36)	
MEP Q1	ref	ref		ref	ref	
MEP Q2	−0.14 (−1.82, 1.53)	−0.54 (−1.52, 0.45)		−0.08 (−1.38, 1.23)	−0.14 (−1.00, 0.72)	
MEP Q3	−0.22 (−1.24, 0.80)	−0.50 (−1.82, 0.82)		0.40 (−0.63, 1.44)	−0.09 (−1.02, 0.85)	
MEP Q4	−0.22 (−1.47, 1.03)	−0.06 (−1.22, 1.10)		0.13 (−1.07, 1.32)	0.59 (−0.43, 1.62)	
MiBP^e^			<0.01			0.04
Sex Q1	ref	1.60 (0.56, 2.64)		ref	1.17 (0.29, 2.04)	
MiBP Q1	ref	ref		ref	ref	
MiBP Q2	0.46 (−0.68, 1.59)	−1.12 (−2.22, −0.02)		0.31 (−0.66, 1.29)	−0.97 (−1.90, −0.03)	
MiBP Q3	0.91 (−0.27, 2.09)	−1.90 (−3.57, −0.23)		0.12 (−0.96, 1.20)	−1.68 (−2.93, −0.43)	
MiBP Q4	−0.09 (−1.38, 1.21)	−1.21 (−2.51, 0.08)		−0.63 (−1.87, 0.60)	−1.17 (−2.38, 0.03)	
	*CGA*			*CYP11A1*		
MnBP^a^			0.01			0.19
Sex Q1	ref	1.95 (0.87, 3.03)		ref	1.03 (0.39, 1.67)	
MnBP Q1	ref	ref		ref	ref	
MnBP Q2	−0.12 (−1.46, 1.22)	−1.59 (−2.87, −0.31)		−0.39 (−1.11, 0.33)	−0.91 (−1.81, −0.02)	
MnBP Q3	0.59 (−0.75, 1.93)	-2.01 (−3.55, −0.48)		−0.23 (−1.12, 0.65)	−1.04 (−1.98, −0.10)	
MnBP Q4	0.16 (−1.18, 1.50)	−1.60 (−3.31, 0.11)		0.15 (−0.78, 1.08)	−0.98 (−2.03, 0.07)	
MBzP^b^			0.05			0.03
Sex Q1	ref	0.92 (−0.28, 2.11)		ref	0.50 (−0.24, 1.24)	
MBzP Q1	ref	ref		ref	ref	
MBzP Q2	−0.43 (−1.61, 0.75)	0.15 (−1.21, 1.50)		−0.28 (−1.00, 0.43)	0.47 (−0.38, 1.32)	
MBzP Q3	0.99 (−0.33, 2.30)	0.30 (−1.01, 1.60)		0.59 (−0.24, 1.42)	0.54 (−0.40, 1.48)	
MBzP Q4	0.68 (−0.67, 2.03)	−0.91 (−2.25, 0.42)		0.96 (0.11, 1.82)	−0.05 (−0.95, 0.85)	
MEHP^c^			0.02			0.11
Sex Q1	ref	0.75 (−0.62, 2.12)		ref	0.59 (−0.21, 1.39)	
MEHP Q1	ref	ref		ref	ref	
MEHP Q2	−0.44 (−1.80, 0.93)	−0.15 (−1.41, 1.11)		−0.32 (−1.08, 0.44)	−0.51 (−1.34, 0.32)	
MEHP Q3	0.13 (−1.11, 1.36)	−1.48 (−2.62, −0.35)		−0.20 (−0.98, 0.58)	−1.10 (−1.91, −0.29)	
MEHP Q4	0.00 (−1.38, 1.38)	0.21 (−1.02, 1.45)		−0.45 (−1.35, 0.44)	−0.08 (−0.90, 0.73)	
MEP^d^			0.38			0.90
Sex Q1	ref	0.72 (−0.52, 1.95)		ref	0.45 (−0.46, 1.36)	
MEP Q1	ref	ref		ref	ref	
MEP Q2	−0.10 (−1.61, 1.41)	−1.20 (−2.38, −0.01)		−0.13 (−1.09, 0.83)	−0.26 (−1.11, 0.59)	
MEP Q3	−0.04 (−1.23, 1.16)	−0.44 (−1.77, 0.88)		0.17 (−0.62, 0.95)	−0.02 (−0.88, 0.84)	
MEP Q4	−0.32 (−1.52, 0.89)	0.12 (−1.31, 1.54)		0.38 (−0.60, 1.35)	0.54 (−0.41, 1.49)	
MiBP^e^			<0.01			0.02
Sex Q1	ref	2.09 (1.12, 3.07)		ref	1.08 (0.44, 1.71)	
MiBP Q1	ref	ref		ref	ref	
MiBP Q2	0.57 (−0.68, 1.82)	−1.71 (−2.94, −0.49)		0.12 (−0.58, 0.83)	−1.02 (−1.84, −0.19)	
MiBP Q3	0.42 (−0.81, 1.66)	−2.48 (−3.79, −1.17)		0.01 (−0.74, 0.76)	−1.34 (−2.24, −0.45)	
MiBP Q4	−0.25 (−1.57, 1.06)	−2.08 (−3.49, −0.68)		−0.29 (−1.16, 0.59)	−0.89 (−1.90, 0.12)	

Log_e_ unit change (beta coefficient, confidence interval) in placental gene expression for an increase in quartile of maternal urinary phthalate in female and male placentas

*Abbreviations*: *MnBP* Mono-n-butyl phthalate, *MBzP* Monobenzyl phthalate, *MEHP* Mono-2-ethylhexyl phthalate, *MEP* Monoethyl phthalate, *MiBP* Mono-iso-butyl phthalate, *MCPP* Mono-3-carboxypropyl phthalate, *CYP19A1,* Cytochrome P450 family 19 subfamily A member 1 (Aromatase), *AHR* Aryl hydrocarbon receptor, *CGA* Chorionic gonadotropin alpha, *CYP11A1* Cytochrome P450 family 11 subfamily A member 1, *Q1* Quartile 1, *Q2* Quartile 2, *Q3* Quartile 3, *Q4* Quartile 4, *ref* referent

^a^MnBP models were adjusted for MBzP, MEHP, MEP. Upper quartile limits for MnBP: 95 nM/l, 174 nM/l, 357 nM/l, 1724 nM/l

^b^MBzP models were adjusted for MnBP, MEHP, MEP. Upper quartile limits for MBzP: 23 nM/l, 60 nM/l, 139 nM/l, 2138 nM/l

^c^MEHP models were adjusted for MnBP, MBzP, MEP. Upper quartile limits for MEHP: 6.1 nM/l, 21 nM/l, 47 nM/l, 744 nM/l

^d^MEP models were adjusted for MnBP, MBzP, MEHP. Upper quartile limits for MEP: 353 nM/l, 735 nM/l, 1708 nM/l, 31 μM/l

^e^MiBP models were adjusted for MBzP, MEHP, MEP. Upper quartile limits for MiBP: 27 nM/l, 52 nM/l, 95 nM/l, 1685 nM/l

In Additional file 1: Table S1, we present associations of the oxidative metabolites and the 4 mRNAs that differed by sex. DEHP-oxo was more strongly associated than MEHP with *CYP19A1* (*p* = 0.09 vs. *p* = 0.46), *AHR* (*p* = 0.09 vs. *p* = 0.90), and *CYP11A1* (*p* = 0.02 vs. *p* = 0.11), based on the p-value for the sex interaction. In male placentas, the DEHP-oxo and *CYP19A1* and *CGA* associations were strongly inverse. In female placentas, the direction of the DEHP-oxo and mRNA association switched direction as compared to MEHP. The oxidative metabolite MCPP was generally less strongly associated with the mRNAs than the monoesters MiBP and MnBP.

Relative to MiBP, urinary MnBP was higher by 15% (95% CI: −4, 33, p-interaction = 0.03) in LGA pregnancies vs. non-LGA pregnancies (Table 3). The oxidative metabolite, MCPP, was elevated by 24% (95% CI: 8, 60) in the LGA pregnancies. MEHP, DEHP-oxo, MBzP, and MEP were not associated with either outcome.

**Table 3:** Z-score differences in urinary phthalates between pregnancies with and without complications

Model	Parameter	Large for gestational age (LGA)	Gestational diabetes mellitus (GDM)
1.1	Intercept	−1.69 (−2.18, −1.21)	−1.45 (−1.86, −1.05)
	β (mean)	0.11 (−0.10, 0.33) p = 0.30	0.23 (0.00, 0.46) p = 0.05
1.2	β (MnBP)	0.25 (−0.06, 0.55)	0.26 (−0.03, 0.55)
	β (MiBP)	−0.02 (−0.23, 0.19)	0.20 (−0.05, 0.44)
		p (interact) = 0.03*	p (interact) = 0.75
1.3	Female β (mean)	−0.09 (−0.39, 0.21)	0.22 (−0.08, 0.52)
	Male β (mean)	0.27 (−0.01, 0.55)	0.24 (−0.10, 0.58)
		p (interact) = 0.08	p (interact) = 0.95
2.1	Intercept	−1.43 (−1.77, −1.08)	−1.62 (−2.00, −1.24)
	β (mean)	0.12 (−0.05, 0.29) p = 0.18	0.11 (−0.08, 0.32) p = 0.27
2.2	β (ΣDEHP oxidative)	−0.11 (−0.28, 0.07)	−0.01 (−0.24, 0.22)
	β (DBP oxidative)	0.34 (0.11, 0.57)*	0.25 (0.00, 0.49)*
		p (interact) < 0.0001	p (interact) = 0.05
2.3	Female β (mean)	0.07 (−0.14, 0.27)	−0.02 (−0.31, 0.26)
	Male β (mean)	0.16 (−0.10, 0.42)	0.25 (−0.01, 0.51)
		p (interact) = 0.60	p (interact) = 0.16
3.1	Intercept	−1.18 (−1.90, −0.46)	−1.23 (−1.93, −0.52)
	β (MBzP)	0.07 (−0.22, 0.37) p = 0.62	−0.27 (−0.59, 0.05) p = 0.10
3.2	Female: β (MBzP)	−0.06 (−0.46, 0.35)	−0.28 (−0.76, 0.19)
	Male: β (MBzP)	0.22 (−0.21, 0.64)	−0.24 (−0.67, 0.19)
		p (interact) = 0.36	p (interact) = 0.60
4.1	Intercept	−0.27 (-0.97, 0.42)	−1.05 (−2.79, 0.69)
	β (MEHP)	−0.26 (−0.59, 0.08) p = 0.14	−0.31 (−0.67, 0.05) p = 0.09
4.2	Female: β (MEHP)	−0.24 (−0.69, 0.22)	−0.35 (−0.87, 0.16)
	Male: β (MEHP)	−0.28 (−0.78, 0.22)	−0.27 (−0.76, 0.21)
		p (interact) = 0.90	p (interact) = 0.53
5.1	Intercept	−0.68 (1.24, −0.12)	−0.80 (−1.33, −0.62)
	β (MEP)	0.05 (−0.28, 0.38) p = 0.77	0.02 (−0.33, 0.38) p = 0.89
5.2	Female: β (MEP)	0.14 (−0.33, 0.62)	0.17 (−0.33, 0.68)
	Male: β (MEP)	−0.02 (−0.47, 0.42)	−0.11 (−0.59, 0.37)
		p (interact) = 0.29	p (interact) = 0.16

Beta coefficients (z-scores, 95% confidence intervals) represent the difference in urinary phthalate concentrations between women with and without pregnancy complications

*Abbreviations*: *MnBP* Mono-n-butyl phthalate, *MBzP* Monobenzyl phthalate, *MEHP* Mono-2-ethylhexyl phthalate, *MEP* Monoethyl phthalate, *MiBP* Mono-iso-butyl phthalate, *MCPP* Mono-3-carboxypropyl phthalate, *DEHP* di-2-ethylhexyl phthalate, *DBP* DiButyl phthalate

^*a*^All models were adjusted for urinary dilution and placental-fetal sex. In Model 1, monoester metabolites of di-n-butyl and di-iso-butyl phthalate were modeled as a correlated vector, with adjustment for log urinary concentrations of MBzP, MEHP, and MEP. In Model 2, the sum of DEHP oxidative metabolites (MEOHP, MEHHP, MECPP) and the DBP oxidative metabolite MCPP were modeled as a correlated vector, with adjustment for urinary concentrations of MnBP, MiBP, MBzP, MEHP, and MEP. In Models 3–5, each metabolite was modeled separately and adjusted for log urinary concentrations of MnBP, MBzP, MEHP, and MEP

Model specific covariates

LGA: Model 1 (MnBP/MiBP): year of birth, season of birth, parity, weight gain (tertiles); Model 2 (oxidative metabolites): year of sample, body mass index at the start of pregnancy (tertiles). Model 3 (MBzP): maternal age, weight at the start of pregnancy, education; Model 4 (MEHP): weight at the start of pregnancy, year of birth; Model 5 (MEP): body mass index at the start of pregnancy (tertiles), parity, household income

GDM: Model 1 (MnBP/MiBP): year of birth, season of birth, parity; Model 2 (oxidative metabolites): year of sample, season of birth; Model 3 (MBzP): maternal age, weight at the start of pregnancy; Model 4 (MEHP): weight at the start of pregnancy, year of birth, maternal age, second hand cigarette smoke, elective abortions; Model 5 (MEP): parity, household income. *p < =0.05

We applied a similar method to estimate associations of the outcomes with correlated vectors of mRNAs. We grouped the mRNAs into those that were associated with phthalates differently in males and females (*CYP19, CYP11A1, CGA*), and those that were not (*PPARG, AHR, HSD17B1, SLC27A4*) (Additional file 3: Table S3, Fig. 2). There were only 3 female vs. 11 male LGA pregnancies in the placenta substudy (Chisq *p* = 0.02). The LGA placentas had 1.5-fold lower (95% CI: −2.8, −1.1) expression of the mRNAs: *PPARG*, *AHR*, *HSD17B1* and *SLC27A4*. The two groups of mRNAs in GDM placentas were lower in male vs. female births. According to mRNA-specific calculations, *CGA* was lower in LGA placentas, but did not differ in GDM vs. non-GDM placentas. We did not observe associations of phthalates or mRNAs with small for gestational age placentas (8% of female births, 6% of male births).

## Discussion

In the course of this study, we made important observations that have informed our understanding of the human placental response to phthalate exposure. First, associations, in some cases, differed by the sex of the placenta. This was non-intuitive as the placenta anatomically, morphologically and functionally does not differ by sex. From an evolutionary point of view, it is also non-intuitive [48]. If the placenta performs markedly better or worse in response to environmental stress in one sex or another, we postulate that his could result in an imbalanced sex ratio and lower reproductive fitness. Secondly, we found that the gene for a key placental hormone hCG that is involved in most physiologic processes in pregnancy [49] and with normal sex differentiation of the male fetus [22], may be a target of phthalate exposure. *CGA*, one of the genes that encodes hCG, was associated with the greatest number of phthalate metabolites. Thirdly, we observed a small number of associations of these gene expression biomarkers and the urinary phthalates with outcomes at birth potentially related to obesogenic actions and/or endocrine functions of the placenta. Taken together, this argues for more consideration and efforts to measure and model the molecular response of the placenta as mediator of the effects of phthalate exposures on the short-term outcome of the pregnancy and the long-term health of the child.

The sex imbalance in the phthalate associations that we measured might be due to sampling variability, or the slightly greater size of male placentas at birth. It may also relate to the general hypothesis that male fetuses and placentas are more susceptible to adversity in utero [50, 51]. The mRNA levels (*HSD17B1, CYP19A1, CGA, PPARG*) were generally higher in males vs. females at the lowest quartile of phthalate, and those differences were either lost or reversed over the range of phthalates. mRNA levels were inversely correlated with phthalates in male placentas. Associations in female placentas were generally positive and significant in the case of high MBzP concentrations. This is similar to trends identified between placental concentrations of brominated flame retardants and placental thyroid hormones [52]. We do not infer from our finding that MBzP is toxic in one sex (i.e. lower gene expression) and beneficial (i.e. higher gene expression) in another, but instead postulate that the mechanisms of phthalate action may differ in the two sexes. Similarly, mechanisms that regulate hormone synthesis may differ between male and female placentas. In this study, correlations between exposure, placental mediators, and birth outcomes were generally stronger in males than females. However, in subsequent studies where we have dosed placental tissue with phthalates, the molecular response of the female was stronger (Adibi et al. in review). In a recently published longitudinal analysis, urinary MiBP was lower in preeclamptic female pregnancies vs. controls [53]. For the DEHP metabolites, investigators observed positive relative risks that were stronger in relation to phthalates measured later vs. earlier in pregnancy. Both sets of findings require caution in interpretation, given the possibility that the urinary metabolite levels might be a result rather than a cause of the placental pathology. Similar to our study, effect sizes for the monoester metabolites were modest (i.e. less than 2-fold), and confidence intervals were wide given the challenge of studying rare outcomes in a cohort setting.

MnBP and MCPP levels in this study were higher in the mothers of babies born large for gestational age. This is consistent with the idea that phthalate exposure in early pregnancy can alter fetal/placental metabolic programming, and the finding that prenatal MCPP in these same pregnancies was associated with 2-fold higher risk of obesity from 4 to 7 years of life [26]. Even though we and others treat MCPP as a metabolite of DBP, we do not know its primary source, why it is rising in the population, and whether its biologic action resembles the monoesters [7, 40, 41]. Phthalates such as MCPP, MnBP and MiBP may be working through pro-obesogenic [54], lipid transport [55], or glucose metabolism mechanisms [56] to increase fetal body mass at any time point in pregnancy. In our data, all quartiles of MnBP and the third quartiles of MiBP and MCPP were strongly associated with decreased levels of placental *PPARG* in male placentas, a transcriptional regulator of adipogenesis that has been implicated in obesogenic effects of endocrine disruptors [57]. Male *PPARG* mRNA was also lower in LGA cases vs. non-cases. In another birth cohort, first trimester MCPP concentrations were associated with circulating levels of leptin in male fetuses [58]. These findings support the idea of a phthalate-induced, sex-specific effect on PPARγ-regulated metabolic programming, possibly a shared effect between the placenta and the fetus [59]. Another interesting parallel between our study and one of the follow-up studies on fat mass is that we both observed significant sex interactions for MnBP and MiBP in which the beta coefficients were positive for females and negative for males, but did not reach significance in the females [27]. Similar to our study, they saw stronger relationships with the non-DEHP metabolites when analyzed as a joint exposure.

To address the within-person, autocorrelation in urinary and placental biomarkers, we implemented an approach of combining these features as a correlated vector, after transformation to a z-score. Even though combined as a correlated vector, we still had the ability to estimate the individual effects of each metabolite or each mRNA.

The significance of phthalate metabolism and elimination as a potential important confounder or mediator of urinary concentrations and placental/birth outcomes remains largely unstudied, and not well addressed in current modeling methods. Originally the urinary monoesters were proposed as a proxy for phthalate diester exposure [60]. Soon after, the oxidative metabolites of DEHP were proposed as a better proxy given higher abundance in urine and longer half-life [61]. Placental mRNAs, measured as primary endpoints in this study, may also be involved in phthalate metabolism and the production of oxidative metabolites [62]. For this reason, we decided to focus our analysis on the monoesters for greater precision in the diester:monoester exposure assessment. Interestingly, the DEHP-oxo had stronger sex interactions than MEHP in relation to the CYP450 enzymes *CYP19A1* and *CYP11A1* and the *AHR*, all genes involved in xenobiotic metabolism and in steroidogenesis. One interpretation is that the CYP450 enzymes contributed to processes downstream of monoester formation and may be less representative of the monoester biologic actions and more representative of non-specific placental metabolism and function. In the absence of empirical knowledge of how the monoester and oxidative metabolites compare in their direct vs. indirect effects on developmental pathways in the placenta, we erred on the side of caution in our modeling strategy.

A limitation of the study was the fact that our sample size was too small to formally test the placental mediation hypothesis of exposure and outcome, especially in the setting of interactions with sex. Parameter estimates may be imprecise due to sample size and due to the difficulties in quantifying mRNA levels in a clinical study using high-throughput methods as we have done here. Given that we had to employ field study methods to sample these placentas at 3 hospitals, we could not maintain tight control on the time elapsed between delivery and tissue sampling. We collected information on time and controlled for it in the analysis. Another limitation was reliance on the birth record for our outcomes and inability to confirm these diagnoses according to physiologic parameters. To assess exposure, we had to rely on a single urine sample collected in the third trimester which can be adequate for MnBP, MBzP and MiBP (intraclass correlation coefficient [ICC] >0.5) but less optimal for MEHP, MEOHP, MEHHP, MECPP, MEP and MCPP (ICC < 0.5) [3, 63].

## Conclusions

We report evidence of sex-specific associations of phthalate exposure and placental gene expression at the level of single genes involved in hCG (and other placental hormone) synthesis and regulation (*CGA, CYP19A1, CYP11A1*), adipogenesis and metabolic programming (*PPARG*), xenobiotic sensing (*AHR*), trophoblast differentiation (*PPARG, CGA*), lipid transport (*PPARG*). We presented additional support for this finding at the level of the secreted hCG protein early in pregnancy [20]. In a companion manuscript, we report partial confirmation of the findings reported here using in vitro models of the early human placenta (Adibi et al. in review). These findings are a first step towards establishing a biologic basis for the associations reported between these same prenatal concentrations and developmental outcomes at 3,5,7, and 8 years of life. Future studies will consider these placental biomarkers as mediators of prenatal phthalate concentrations and developmental outcomes in the children.

## Additional files

Additional file 1: Table S1. Associations of placental gene expression and maternal urinary oxidative metabolites of DEHP and DBP. (DOCX 38 kb)

Additional file 2: Table S2. Associations of placental gene expression in female and male placentas with maternal urinary monoester phthalates. (DOCX 29 kb)

Additional file 3: Table S3. Z-score differences in placental mRNAs between pregnancies with and without complications. (DOCX 76 kb)

## Abbreviations

*CGA*: Chorionic gonadotropin alpha
CI: Confidence interval
*CYP19A1*: Cytochrome P450 family 19 subfamily A member 1
DEHP: Di-2-ethylhxyl phthalate
GDM: Gestational diabetes mellitus
hCG: Human chorionic gonadotropin
LGA: Large for gestational age
MBzP: Monobenzyl phthalate
MCPP: Mono-3-carboxypropyl phthalate
MECPP: Mono-2-ethyl-5-carboxypentyl phthalate
MEHHP: Mono-2-ethyl-5-hydroxyhexyl phthalate
MEHP: Mono-2-ethylhexyl phthalate
MEOHP: Mono-2-ethyl-5-oxohexyl phthalate
MEP: Monoethyl phthalate
MiBP: Mono-iso-butyl phthalate
MnBP: Mono-n-butyl phthalate
*PPARG*: Peroxisome proliferator activated receptor protein gamma
