# Supplementary material for: Maternal urinary phthalates and sex-specific placental mRNA levels in an urban birth cohort
Source: Environ Health. 2017 Apr 5;16:35. doi: 10.1186/s12940-017-0241-5 (PMC5382502; doi:10.1186/s12940-017-0241-5)
Supplement: Supplementary file 1 — Associations of placental gene expression and maternal urinary oxidative metabolites of DEHP and DBP. (DOCX 38 kb) [file 12940_2017_241_MOESM1_ESM.docx]

Additional file 1: Table S1. Associations of placental gene expression and maternal urinary oxidative metabolites of DEHP and DBP.

|  | β (Females)  (95% CI) | β (Males)  (95% CI) | | Sex by phthalate interaction  *P*-value | | β (Females)  (95% CI) | β (Males)  (95% CI) | | Sex by phthalate interaction  *P*-value | |
| --- | --- | --- | --- | --- | --- | --- | --- | --- | --- | --- |
|  | *CYP19A1* | | | | | *AHR* | | | | |
| ΣDEHP oxidative^a^ | | |  | | 0.09 |  | |  | | 0.09 |
| Sex Q1 | ref | | 1.33 (0.21, 2.44) | |  | ref | | 1.33 (0.45, 2.20) | |  |
| Q1 | ref | | ref | |  | ref | | ref | |  |
| Q2 | -0.02 (-1.33, 1.28) | | -0.83 (-1.98, 0.31) | |  | 0.14 (-0.96, 1.24) | | -0.53 (-1.48, 0.43) | |  |
| Q3 | 0.53 (-0.87, 1.92) | | -1.45 (-2.83, -0.07) | |  | 1.30 (-0.18, 2.77) | | -0.56 (-1.73, 0.60) | |  |
| Q4 | -0.86 (-2.38, 0.66) | | -1.92 (-3.36, -0.48) | |  | -0.02 (-1.36, 1.33) | | -1.20 (-2.51, 0.11) | |  |
| DBP oxidative^b^ | | |  | | 0.03 |  | |  | | 0.17 |
| Sex Q1 | ref | | 1.02 (-0.07, 2.11) | |  | ref | | 0.78 (0.01, 1.56) | |  |
| MCPP Q1 | ref | | ref | |  | ref | | ref | |  |
| MCPP Q2 | 0.04 (-1.12, 1.20) | | 0.13 (-0.85, 1.11) | |  | 0.08 (-1.00, 1.16) | | 0.24 (-0.54, 1.02) | |  |
| MCPP Q3 | 0.45 (-0.74, 1.63) | | -1.26 (-2.38, -0.15) | |  | -0.05 (-1.00, 0.90) | | -1.11 (-1.95, -0.27) | |  |
| MCPP Q4 | 0.67 (-0.49, 1.84) | | -0.48 (-1.80, 0.84) | |  | 0.29 (-0.71, 1.28) | | -0.47 (-1.51, 0.56) | |  |
|  | *CGA* | | | | | *CYP11A1* | | | | |
| ΣDEHP oxidative^a^ | | |  | | 0.04 |  | |  | | 0.02 |
| Sex Q1 | ref | | 1.58 (0.50, 2.66) | |  | ref | | 1.01 (0.35, 1.67) | |  |
| Q1 | ref | | ref | |  | ref | | Ref | |  |
| Q2 | -0.45 (-1.72, 0.83) | | -1.14 (-2.47, 0.19) | |  | -0.001 (-0.85, 0.85) | | -0.35 (-1.19, 0.49) | |  |
| Q3 | 0.16 (-1.32, 1.63) | | -2.27 (-3.63, -0.92) | |  | 0.44 (-0.47, 1.35) | | -1.05 (-1.96, -0.15) | |  |
| Q4 | -1.57 (-3.09, -0.05) | | -2.33 (-3.98, -0.68) | |  | -0.67 (-1.74, 0.40) | | -0.99 (-2.14, 0.15) | |  |
| DBP oxidative^b^ | | |  | | 0.11 |  | |  | | 0.19 |
| Sex Q1 | ref | | 1.29 (0.22, 2.35) | |  | ref | | 0.72 (0.10, 1.35) | |  |
| MCPP Q1 | ref | | ref | |  | ref | | ref | |  |
| MCPP Q2 | -0.09 (-1.34, 1.17) | | -0.17 (-1.28, 0.94) | |  | -0.08 (-0.89, 0.72) | | 0.14 (-0.57, 0.84) | |  |
| MCPP Q3 | 0.57 (-0.74, 1.88) | | -1.13 (-2.48, 0.22) | |  | -0.02 (-0.81, 0.76) | | -0.86 (-1.70, -0.02) | |  |
| MCPP Q4 | 0.94 (-0.39, 2.27) | | -0.20 (-1.58, 1.18) | |  | 0.50 (-0.41, 1.41) | | -0.01 (-0.86, 0.84) | |  |
| Abbreviations: MCPP, Mono-3-carboxypropyl phthalate; DEHP, Di-2-ethylhexyl phthalate; *CYP19A1,* Cytochrome P450 family 19 subfamily A member 1 (Aromatase); *AHR*, Aryl hydrocarbon receptor; *CGA*, Chorionic gonadotropin alpha; *CYP11A1*, Cytochrome P450 family 11 subfamily A member 1; Q1, Quartile 1; Q2, Quartile 2; Q3, Quartile 3; Q4, Quartile 4; ref, referent; DEHP, di-2-ethylhexyl phthalate. MEHHP, Mono-2-ethyl-5-hydroxyhexyl phthalate; MEOHP, Mono-2-ethyl-5-oxohexyl phthalate; MECPP, Mono-2-ethyl-5-carboxypentyl; DBP, Di-butyl phthalate; MCPP, Mono-3-carboxy-propyl phthalate.  Log_e_ unit change (beta coefficient, confidence interval) in placental gene expression for an increase in quartile of maternal urinary phthalate in female and male placentas.  ^a^ ΣDEHP oxidative=(MEHHP*(1/294))+MEOHP*(1/292))+(MECPP*(1/308)) x 1000 (nmol/L). Models were adjusted for MnBP, MiBP, MBzP, MEHP, MEP, MCPP. Upper quartile limits for DEHP oxidative: 134 nM/l, 251 nM/l, 502 nM/l, 6879 nM/l.  ^b^ DBP oxidative=MCPP. Models were adjusted for MnBP, MiBP, MBzP, MEHP, MEP and DEHP oxidative metabolites. Upper quartile limits for MCPP: 4.4 nM/l, 7.9 nM/l, 14 nM/l, 106 nM/l. | | | | | | | | | | |
