# Supplementary material for: Maternal urinary phthalates and sex-specific placental mRNA levels in an urban birth cohort
Source: Environ Health. 2017 Apr 5;16:35. doi: 10.1186/s12940-017-0241-5 (PMC5382502; doi:10.1186/s12940-017-0241-5)
Supplement: Supplementary file 2 — Associations of placental gene expression in female and male placentas with maternal urinary monoester phthalates. (DOCX 29 kb) [file 12940_2017_241_MOESM2_ESM.docx]

Additional file 2: Table S2. Associations of placental gene expression in female and male placentas with maternal urinary monoester phthalates.

|  | β (Females)  (95% CI) | β (Males)  (95% CI) | Sex by phthalate interaction  *P*-value | β (Females)  (95% CI) | β (Males)  (95% CI) | Sex by phthalate interaction  *P*-value |
| --- | --- | --- | --- | --- | --- | --- |
|  | *HSD17B1* | | | *SLC27A4* | | |
| MnBP^a^ |  |  | 0.33 |  |  | 0.30 |
| Sex Q1 | ref | 0.36 (-0.31, 1.03) |  | ref | 0.39 (-0.40, 1.18) |  |
| MnBP Q1 | ref | ref |  | ref | ref |  |
| MnBP Q2 | 0.63 (-0.18, 1.45) | 0.40 (-0.52, 1.31) |  | 0.61 (-0.26, 1.49) | -0.58 (-1.59, 0.42) |  |
| MnBP Q3 | 0.53 (-0.45, 1.52) | 0.45 (-0.71, 1.61) |  | 0.38 (-0.58, 1.35) | -0.29 (-1.33, 0.74) |  |
| MnBP Q4 | 1.24 (0.25, 2.23) | 0.32 (-0.97, 1.61) |  | 0.22 (-0.60, 1.05) | -0.14 (-1.28, 1.01) |  |
| MBzP^b^ |  |  | 0.17 |  |  | 0.41 |
| Sex Q1 | ref | 0.71 (-0.03, 1.45) |  | ref | 0.33 (-0.46, 1.11) |  |
| MBzP Q1 | ref | ref |  | ref | ref |  |
| MBzP Q2 | -0.09 (-0.88, 0.71) | -0.81 (-1.77, 0.16) |  | 0.21 (-0.58, 1.00) | -0.21 (-1.16, 0.74) |  |
| MBzP Q3 | 0.57 (-0.36, 1.49) | -0.34 (-1.34, 0.66) |  | 0.49 (-0.44, 1.41) | -0.54 (-1.49, 0.41) |  |
| MBzP Q4 | 0.30 (-0.62, 1.23) | -0.80 (-1.83, 0.23) |  | 0.09 (-0.84, 1.02) | -0.43 (-1.39, 0.54) |  |
| MEHP^c^ |  |  | 0.98 |  |  | 0.70 |
| Sex Q1 | ref | -0.06 (-0.78, 0.67) |  | ref | -0.12 (-0.82, 0.57) |  |
| MEHP Q1 | ref | ref |  | ref | ref |  |
| MEHP Q2 | 0.27 (-0.49, 1.02) | 0.37 (-0.46, 1.20) |  | -0.53 (-1.31, 0.24) | -0.28 (-1.11, 0.55) |  |
| MEHP Q3 | -0.19 (-0.91, 0.53) | 0.04 (-0.92, 1.00) |  | 0.09 (-0.81, 0.99) | 0.09 (-0.75, 0.93) |  |
| MEHP Q4 | 0.05 (-0.81, 0.90) | 0.09 (-0.62, 0.79) |  | 0.32 (-0.48, 1.11) | -0.11 (-0.88, 0.65) |  |
| MEP^d^ |  |  | 0.91 |  |  | 0.73 |
| Sex Q1 | ref | 0.28 (-0.55, 1.10) |  | ref | 0.02 (-0.86, 0.90) |  |
| MEP Q1 | ref | ref |  | ref | ref |  |
| MEP Q2 | 0.13 (-0.75, 1.02) | -0.13 (-0.93, 0.68) |  | -0.29 (-1.23, 0.66) | -0.20 (-1.00, 0.61) |  |
| MEP Q3 | -0.15 (-0.94, 0.64) | -0.58 (-1.45, 0.30) |  | 0.07 (-0.73, 0.86) | -0.53 (-1.52, 0.46) |  |
| MEP Q4 | 0.21 (-0.73, 1.15) | -0.05 (-0.98, 0.87) |  | 0.14 (-0.83, 1.12) | -0.08 (-0.97, 0.81) |  |
| MiBP^e^ |  |  | 0.24 |  |  | 0.68 |
| Sex Q1 | ref | 0.57 (0.02, 1.12) |  | ref | 0.07 (-0.76, 0.89) |  |
| MiBP Q1 | ref | ref |  | ref | ref |  |
| MiBP Q2 | 0.06 (-0.80, 0.93) | -0.69 (-1.54, 0.16) |  | -0.20 (-1.02, 0.62) | -0.33 (-1.32, 0.66) |  |
| MiBP Q3 | 0.34 (-0.42, 1.11) | -0.68 (-1.63, 0.27) |  | 0.39 (-0.62, 1.39) | -0.26 (-1.31, 0.79) |  |
| MiBP Q4 | -0.50 (-1.38, 0.37) | -0.82 (-1.75, 0.11) |  | -0.14 (-1.34, 1.07) | -0.21 (-1.27, 0.86) |  |
|  | *PPARG* | | | *PTGS2^g^* | | |
| MnBP^a^ |  |  | 0.21 |  |  | 0.76 |
| Sex Q1 | ref | 0.69 (0.20, 1.19) |  | ref | 0.07 (-0.90, 1.06) |  |
| MnBP Q1 | ref | ref |  | ref | ref |  |
| MnBP Q2 | -0.27 (-0.98, 0.43) | -0.90 (-1.59, -0.20) |  | -0.15 (-0.98, 0.68) | -0.51 (-1.38, 0.36) |  |
| MnBP Q3 | -0.46 (-1.32, 0.40) | -0.75 (-1.46, -0.03) |  | -0.36 (-1.41, 0.69) | -0.16 (-1.12, 0.80) |  |
| MnBP Q4 | -0.18 (-1.02, 0.67) | -1.07 (-2.02, -0.11) |  | 0.50 (-0.43, 1.43) | 0.16 (-1.07, 1.38) |  |
| MBzP^b^ |  |  | 0.17 |  |  | 0.51 |
| Sex Q1 | ref | 0.00 (-0.64, 0.64) |  | ref | -0.34 (-1.40, 0.72) |  |
| MBzP Q1 | ref | ref |  | ref | ref |  |
| MBzP Q2 | -0.50 (-1.15, 0.15) | 0.42 (-0.34,1.18) |  | -0.50 (-1.43, 0.43) | 0.36 (-0.59, 1.30) |  |
| MBzP Q3 | 0.40 (-0.32, 1.12) | 0.41 (-0.33, 1.14) |  | 0.20 (-0.85, 1.26) | 0.52 (-0.33, 1.37) |  |
| MBzP Q4 | 0.02 (-0.74, 0.77) | 0.02 (-0.80, 0.83) |  | 0.30 (-0.87, 1.47) | 0.25 (-0.70, 1.19) |  |
| MEHP^c^ |  |  | 0.34 |  |  | 0.87 |
| Sex Q1 | ref | 0.04 (-0.56, 0.64) |  | ref | 0.08 (-0.94, 1.09) |  |
| MEHP Q1 | ref | ref |  | ref | ref |  |
| MEHP Q2 | -0.54 (-1.18, 0.09) | 0.02 (-0.57, 0.60) |  | -0.28 (-1.21, 0.65) | -0.20 (-0.92, 0.53) |  |
| MEHP Q3 | -0.23 (-0.89, 0.43) | -0.38 (-1.03, 0.28) |  | -0.01 (-1.23, 1.20) | -0.43 (-1.24, 0.39) |  |
| MEHP Q4 | -0.42 (-1.17, 0.33) | -0.07 (-0.64, 0.50) |  | 0.04 (-0.96, 1.05) | -0.13 (-0.90, 0.65) |  |
| MEP^d^ |  |  | 0.12 |  |  | 0.41 |
| Sex Q1 | ref | 0.55 (-0.20, 1.29) |  | ref | -0.01 (-1.08, 1.05) |  |
| MEP Q1 | ref | ref |  | ref | ref |  |
| MEP Q2 | 0.39 (-0.47, 1.25) | -0.47 (-1.10, 0.17) |  | 0.06 (-1.13, 1.24) | -0.37 (-0.95, 0.21) |  |
| MEP Q3 | 0.66 (-0.11, 1.42) | 0.16 (-0.50, 0.82) |  | 0.03 (-1.08, 1.14) | -0.35 (-1.14, 0.44) |  |
| MEP Q4 | 0.39 (-0.48, 1.26) | 0.46 (-0.21,1.13) |  | -0.14 (-1.27, 1.00) | 0.24 (-0.60, 1.08) |  |
| MiBP^e^ |  |  | 0.13 |  |  | 0.43 |
| Sex Q1 | ref | 0.53 (0.02, 1.03) |  | ref | 0.07 (-0.85, 0.99) |  |
| MiBP Q1 | ref | ref |  | ref | ref |  |
| MiBP Q2 | -0.34 (-0.97, 0.29) | -0.49 (-1.12, 0.15) |  | -0.73 (-1.73, 0.27) | -0.37 (-1.18, 0.43) |  |
| MiBP Q3 | 0.07 (-0.74, 0.87) | -1.00 (-1.84, -0.15) |  | 0.16 (-0.92, 1.23) | 0.02 (-0.99, 1.04) |  |
| MiBP Q4 | -0.55 (-1.27, 0.18) | -0.69 (-1.44, 0.05) |  | -0.55 (-1.65, 0.54) | -0.94 (-1.94, 0.06) |  |

| Abbreviations: MnBP, Mono-n-butyl phthalate; MBzP, Monobenzyl phthalate; MEHP, Mono-2-ethylhexyl phthalate; MEP, Monoethyl phthalate; MiBP, Mono-iso-butyl phthalate; *HSD17B1*, Hydroxysteroid 17-beta dehydrogenase 1;  *SLC27A4*, Solute carrier family 27 member 4 (FATP4);  *PPARG*, Peroxisome proliferator activated receptor gamma;  *PTGS2*, Prostaglandin-endoperoxide synthase 2; Q1, Quartile 1; Q2, Quartile 2; Q3, Quartile 3; Q4, Quartile 4; ref, referent.  Log_e_ unit change (beta coefficient, confidence interval) in placental gene expression for an increase in quartile of maternal urinary phthalate in female and male placentas.  ^a^ MnBP models were adjusted for MBzP, MEHP, MEP. Upper quartile limits for MnBP: 95 nM/l, 174 nM/l, 357 nM/l, 1724 nM/l;  ^b^ MBzP models were adjusted for MnBP, MEHP, MEP. Upper quartile limits for MBzP: 23 nM/l, 60 nM/l, 139 nM/l, 2138 nM/l;  ^c^ MEHP models were adjusted for MnBP, MBzP, MEP. Upper quartile limits for MEHP: 6.1 nM/l, 21 nM/l, 47 nM/l, 744 nM/l;  ^d^ MEP models were adjusted for MnBP, MBzP, MEHP. Upper quartile limits for MEP: 353 nM/l, 735 nM/l, 1708 nM/l, 31 μM/l;  ^e^ MiBP models were adjusted for MBzP, MEHP, MEP. Upper quartile limits for MiBP: 27 nM/l, 52 nM/l, 95 nM/l, 1685 nM/l;  ^g^*PTGS2* was measured was measured in the second batch and not the pilot analysis (N=124 placentas). |
| --- |
