# Supplementary material for: Maternal urinary phthalates and sex-specific placental mRNA levels in an urban birth cohort
Source: Environ Health. 2017 Apr 5;16:35. doi: 10.1186/s12940-017-0241-5 (PMC5382502; doi:10.1186/s12940-017-0241-5)
Supplement: Supplementary file 3 — Z-score differences in placental mRNAs between pregnancies with and without complications. (DOCX 76 kb) [file 12940_2017_241_MOESM3_ESM.docx]

Additional file 3: Table S3. Z-score differences in placental mRNAs between pregnancies with and without complications.

| Model*^a^* | Parameter | Large for gestational age  (LGA) | Gestational diabetes mellitus  (GDM) |
| --- | --- | --- | --- |
| 1.1 | Intercept | -0.55 (-1.03, -0.07) | -0.51 (-0.91, -0.11) |
|  | β (mean of 3 genes) | -0.32 (-0.66, 0.02), p=0.07 | -0.15(-0.53, 0.22), p=0.41 |
| 1.2 | β (*CYP19*) | -0.35 (-0.81, 0.11) | -0.23 (-0.74, 0.28) |
|  | β (*CYP11A1*) | -0.32 (-0.66, 0.02) | -0.24 (-0.60, 0.13) |
|  | β (*CGA*) | -0.28 (-0.59, 0.02) | 0.04 (-0.30, 0.39) |
|  |  | p (interaction) =0.72 | p (interaction) =0.0007* |
| 1.3 | Female: β (mean of 3 genes) | -0.56 (-1.30, 0.18) | 0.08 (-0.44, 0.60) |
|  | Male: β (mean of 3 genes) | -0.15 (-0.51, 0.21) | -0.28 (-0.73, 0.16) |
|  |  | 0.78 | 0.04* |
| 2.1 | Intercept | -0.25 (-0.70, 0.19) | -0.33 (-0.73, 0.08) |
|  | β (mean of 4 genes) | -0.37 (-0.70, -0.03), p=0.03* | -0.26 (-0.60, 0.08), p=0.13 |
| 2.2 | β (*PPARG*) | -0.45 (-0.84, -0.06)* | -0.20 (-0.63, 0.22) |
|  | β (*AHR*) | -0.48 (-0.87, -0.08)* | -0.35 (-0.75, 0.04) |
|  | β (*HSD17B1*) | -0.27 (-0.77, 0.23) | -0.34 (-0.66, -0.01)* |
|  | β (*SLC27A4*) | -0.36 (-0.69, -0.03)* | -0.32 (-0.77, 0.13) |
|  |  | p (interaction) =0.86 | p (interaction) =0.23 |
| 2.3 | Female: β (mean of 3 genes) | -0.56 (-1.27, 0.14) | 0.05 (-0.35, 0.45) |
|  | Male: β (mean of 3 genes) | -0.27 (-0.63, 0.09) | -0.47 (-0.91, -0.03)* |
|  |  | p (interaction) =0.67 | p (interaction) =0.02* |

Abbreviations: *CYP19A1*, Cytochrome P450 family 19 subfamily A member 1; *AHR*, Aryl hydrocarbon receptor; *CGA*, Chorionic gonadotropin alpha; *CYP11A1*, Cytochrome P450 family 11 subfamily A member 1; *HSD17B1*, Hydroxysteroid 17-beta dehydrogenase 1; *SLC27A4*, Solute carrier family 27 member 4 (FATP4); *PPARG*, Peroxisome proliferator activated receptor gamma; *PTGS2*, Prostaglandin-endoperoxide synthase 2.

Beta coefficients (z-scores, 95% confidence intervals) represent the difference in placental mRNAs between pregnancies with and without pregnancy complications.

*^a^* All models were adjusted for placental-fetal sex, urinary dilution, *RN18S* mRNA (housekeeping gene), body mass index at the beginning of pregnancy, qPCR batch, technician, urinary dilution, quartiles of urinary phthalates (MnBP, MBzP, MEHP, MEP) and year of sample. Model 1 included the mRNAs that were associated with prenatal phthalates in a sex-specific manner. Model 2 included the mRNAs that did not differ by sex in their associations with phthalates.

*p<=0.05
